# Impact of BCG vaccination on incidence of tuberculosis disease in southern Ireland

**DOI:** 10.1186/s12879-019-4026-z

**Published:** 2019-05-09

**Authors:** Eileen Sweeney, Darren Dahly, Nahed Seddiq, Gerard Corcoran, Mary Horgan, Corinna Sadlier

**Affiliations:** 1 Department of Infectious Diseases, Cork University Hospital, T12 DC4A Cork, Ireland; 2 Health Research Board Clinical Research Facility, Department of Epidemiology and Public Health, University College Cork, Cork, Ireland; 3 Department of Microbiology, Cork University Hospital, Cork, Ireland; 4 School of Medicine, University College Cork, Cork, Ireland; 5 Royal College of Physicians of Ireland, Dublin, Ireland

**Keywords:** Bacille Camille-Guerin (BCG), *Mycobacterium tuberculosis* (MTB), Vaccines

## Abstract

**Background:**

Tuberculosis (TB) is the ninth leading cause of death worldwide and the leading cause from a single infectious agent. Bacillus Calmette-Guerin (BCG) is the only licensed vaccine for TB, yet its efficacy remains debated with variations in vaccine sub-strains, policies, and practices observed across the world. Three BCG vaccination policies were implemented across adjoining regions in the South West of Ireland from 1972; neonatal vaccination (vaccinated Region-A), vaccination of children aged 10–12 years (vaccinated Region-B) and no vaccination (unvaccinated Region-C). The aim of this study is to examine the impact of different BCG vaccination policies on incidence of TB disease in the South of Ireland over a 13-year period.

**Methods:**

Cases of active TB disease from 2003 to 2016 were identified through surveillance data. Residential addresses for each case were geocoded using the Google Maps API. Addresses were linked to 2011 census population data and to Local Health Offices BCG coverage data for study regions A-C. A steady-state population was assumed to calculate the 13-year incidence of TB disease. Using SatScan (v9.4.4), spatial clusters were identified at a small area level with the spatial scan statistic based on the discrete Poisson probability distribution.

**Results:**

Of 621 TB disease cases identified, 510 could be linked to the study area based on the reported addresses. The median age was 42 years (range 4 months - 94 years), 65% male and 66% Irish born. The incidence of TB disease was higher in the unvaccinated population, region-C 132/100,000 (95% CI 116–150) versus vaccinated region-A 56/100,000 (95%CI 45–69) and region-B 44/100,000 (95%CI 29–63). A spatial cluster analysis identified a single high-risk cluster in region -C where the relative risk (vs. the areas outside of the cluster) was 4.94 (95% CI 4.03 to 5.96).

**Conclusion:**

Our study demonstrates significant regional variation in the incidence of TB in demographically similar populations based on BCG vaccination policy. This observation is particularly noteworthy in a country with low TB disease incidence such as Ireland. These findings strengthen existing data demonstrating efficacy of BCG vaccination for primary prevention of TB disease.

**Electronic supplementary material:**

The online version of this article (10.1186/s12879-019-4026-z) contains supplementary material, which is available to authorized users.

## Background

Tuberculosis (TB) is the ninth leading cause of death worldwide and the leading cause from a single infectious agent. In 2017 the World Health Organisation (WHO) estimated that 10 million people were infected with *Mycobacterium tuberculosis* (MTB) and that there had been 1.6 million TB related deaths [1]. The discovery of the Bacille Camille-Guerin (BCG) vaccine in the 1920s was a milestone in TB control. In 1949 BCG vaccine was introduced in Ireland by Dr. Dorothy Stopford Price [2].

Indication for and efficacy of BCG vaccine remains debated with variations in vaccine sub-strains, policies, and practices observed across the world [3]. The BCG vaccine has been subject to several trials which have estimated an overall protective efficacy of 60–80% against severe forms of TB disease in children, particularly meningitis [4]. Protection against pulmonary TB varies according to age of administration of BCG vaccine and geographical location. By the 1970s, pilot studies in Western Europe demonstrated a decline in risk of serious forms of TB in children [5].

Data also indicated a protective effect of BCG vaccine in adults, nevertheless, failure to impact the global incidence of TB resulted in a discontinuation of BCG vaccination programmes in several countries including former Czechoslovakia (1961–1972) and Sweden (1975) [6].

In Ireland, universal neonatal BCG vaccination was introduced in the 1950’s. The vaccine was discontinued in a region in the South of Ireland (Cork) in 1972 based on low incidence of TB disease in the area and concerns regarding interpretation of positive Mantoux tests in the immunised population potentially complicating future diagnosis of TB disease [2]. Elsewhere in Ireland, a universal BCG vaccination policy remained in place as evidence continued to support its use in the broader Irish context [7].

BCG vaccination policy in Ireland was reviewed in accordance with other European countries in 2014 [8, 9]. Universal BCG vaccination was discontinued in 2015 as a result of the global BCG vaccine shortage and is no longer recommended in national immunisation guidelines in Ireland as of 2016 [10].

In this study, we analyse TB surveillance data in the South of Ireland over a thirteen-year period (2003–2016). This data is unique in that in compares three different BCG vaccination policies across bordering geographical regions, neonatal vaccination (Region-A), vaccination of children aged 10–12 years (Region-B) and no vaccination (unvaccinated, Region-C).

### Aim

The aim of this study is to examine the impact of three different BCG vaccination policies on observed incidence of TB disease in the South of Ireland over a 13-year period.

### Data collection

All cases of suspected TB disease, whether in the community or hospital setting, have isolates taken that are sent to the regional TB laboratory. Surveillance data from the regional TB laboratory was used to identify all MTB culture isolates from 2003 to 2016. Data were collected over a 13-year period (08/2003 to 12/2016) for all reported cultured cases of MTB.

Cases were divided by Local Health Offices (LHO) region within the South of Ireland (North Lee, South Lee, West Cork, North Cork and Kerry). National crude incidence rates were compared with Health Service Executive (HSE) South rates using census 2011 population data in keeping with HPSC (Health Protection Surveillance Centre) reports [11].

Ethical approval was granted through the Research Ethics Committee of the Cork Teaching Hospitals.

## Methods

### Study design and population

All cases included in our study were exclusively active TB disease. Isolates on all suspected cases of active TB disease within the study area are routinely sent to Cork University Hospital for culturing and identification. The study preceded the availability of conventional molecular methods for identification of TB disease, thus all cases were identified through growth on culture media.

Residential addresses for each TB case were geocoded using the Google Maps API. Information about case locations were spatially linked to 2011 census population data [12] at the small area level using QGIS (v 2.18.13). Spatial data were similarly linked to the coverage of the HSE LHO for Kerry, West Cork, South Lee, North Lee, and North Cork. The 13-year incidence of TB disease was calculated assuming a steady-state population. Using SatScan (v 9.4.4), we identified spatial clusters of higher than normal TB disease incidence at the small area level with the spatial scan statistic based on the discrete Poisson probability distribution.

### Exclusion criteria

All cases of non-tuberculosis mycobacterium or any case of MTB cultured in the regional TB lab but not located in our study area were excluded.

## Results

A total of 621 cases of TB disease were identified during the study period. The median age was 42 years (range 4 months - 94 years; 95% of the sample were older than 18 years of age). The addresses of 577 (93%) cases were successfully geocoded. We excluded 67 cases for linking to addresses outside the coverage area, thus 510 (80%) cases were included in further analysis. 327 (65%) cases were male, 339 (66%) of Irish origin. 398 (78%) cases were pulmonary tuberculosis 104 (20%) cases were extra pulmonary.

**Fig. 1:**
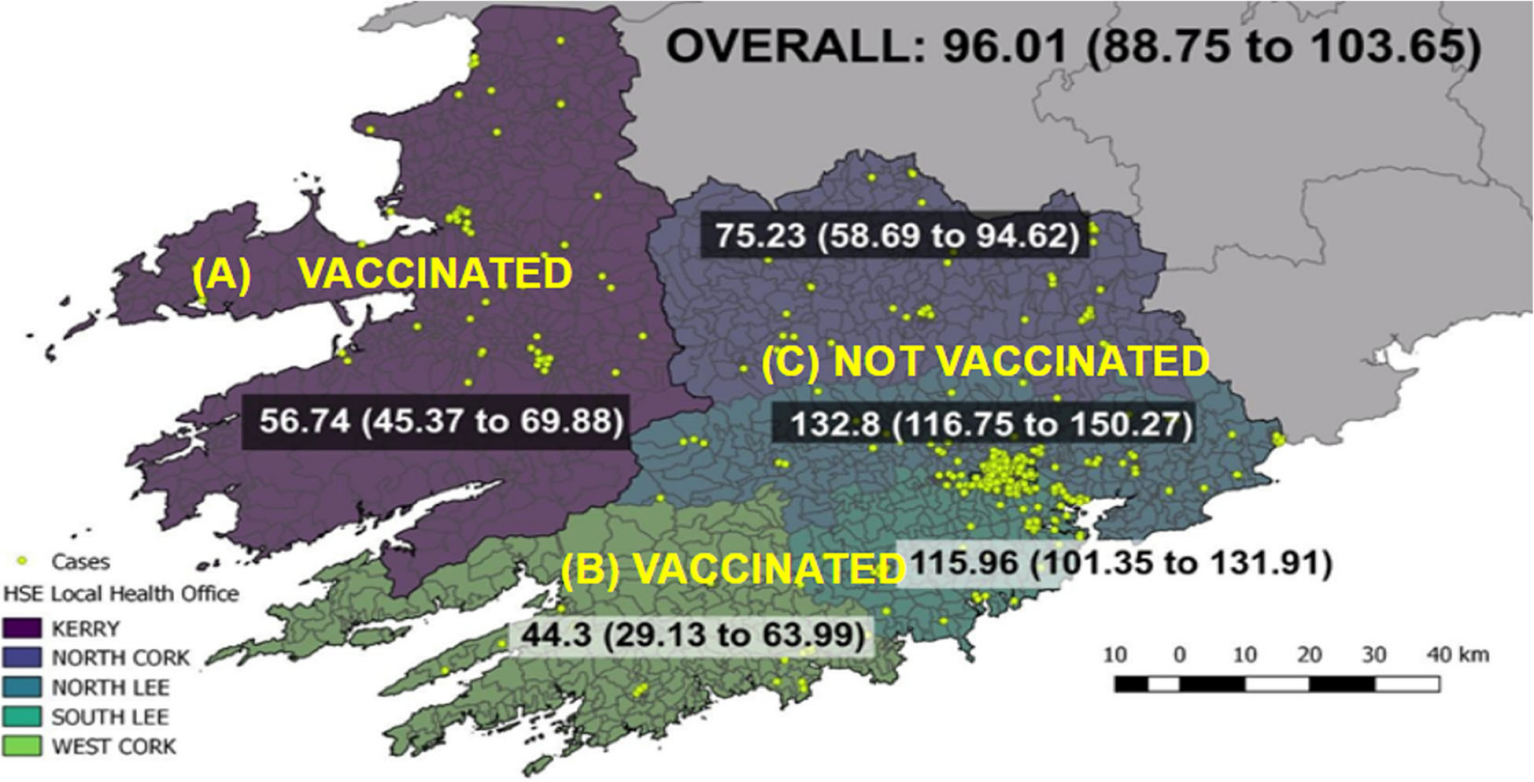
Incidence of Tuberculosis Disease in Southern Ireland per 100,000 [95% CI] from 2003 to 2016. **a** (neonatal vaccination programme in a rural area). **b** (childhood vaccination programme in a rural area). **c** (unvaccinated population in an urban area). Abbreviations: CI Confidence Interval. Attribution: Dr. Darren Dahly, Principal Statistician, Cork University Hospital

The incidence of TB disease in Region-A (neonatal vaccination programme in a rural area) was 56/100,000 (95% confidence interval, 95% CI, 45–69). The incidence of TB disease in Region-B (childhood vaccination programme in a rural area) was 44/100,000 (95% CI, 29–63). The incidence of TB disease in Region-C (unvaccinated population in an urban area) was 132/100,000 (95% CI, 116–150) (Fig. 1).

Within the unvaccinated population (Region-C), 65% of TB cases were Irish born compared to 72% in the vaccinated population (Table 1).

**Table 1:** Demographics of Tuberculosis Disease cases in Southern Ireland, 2003–2016

TB Disease	Region A *	Region B**	Region C ***			Total
	Kerry	West Cork	North Cork	North Lee	South Lee	
Number of Cases / 100,000	66 (13%)	20 (4%)	54 (10.5%)	193 (38%)	177 (34.5%)	510
Median age @ dx	44.5	61.5	43.5	39	39	42
Age </= 18	3	0	2	13	13	31
Age range	0-92 yrs	20-85 yrs	2-89 yrs	0-90 yrs	0-93 yrs	0-93 yrs
Male	44	10	30	131	117	332
Female	22	10	24	59	59	174
Unknown	0	0	0	3	1	4
HIV	1	0	2	2	9	14
Irish born	49	13	39	124	114	339
Foreign	12	6	8	41	34	101
Unknown	5	1	7	28	29	70
MDR	0	0	1	2	1	4

*Neonatal vaccination policy, ** Vaccinated at 12–13 years ***Unvaccinated

Unknown indicates where data was unavailable.

Abbreviations: **MDR** Multi Drug Resistant

A spatial cluster analysis identified a single high-risk cluster in Cork City (unvaccinated population in Region -C). The cluster included 138 cases in a population of 46,000, and the relative risk (vs. the areas outside of the cluster) was 4.94 (95% CI 4.03 to 5.96) (Additional file 1.).

**Fig. 2:**
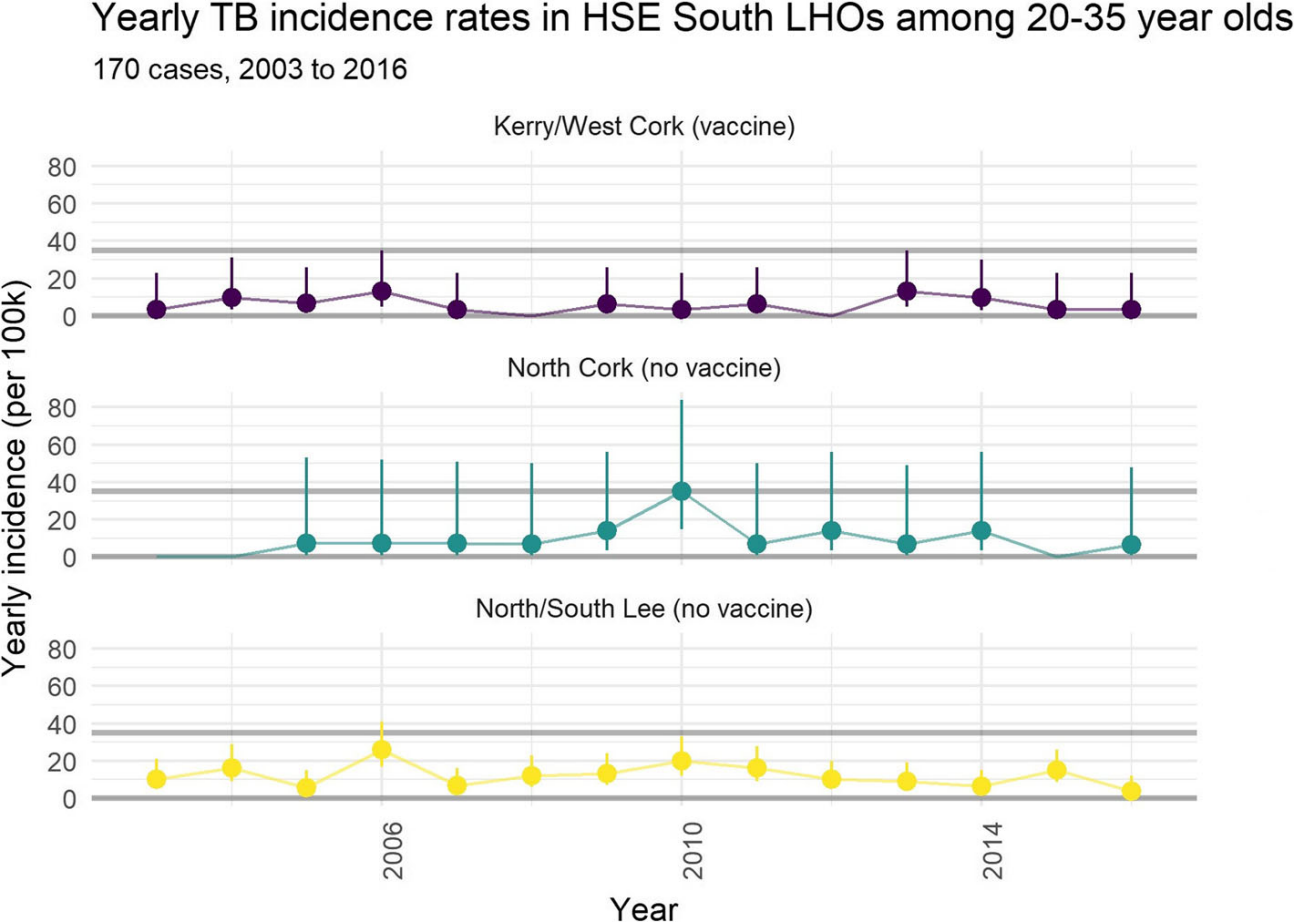
Distribution of Tuberculosis Disease cases in Southern Ireland over Time Numbers plotted: Incidence of TB disease per 100,000 [95% CI] Abbreviations: HSE Health Service Executive, LHO Local Health Office Attribution: Dr. Darren Dahly, Principal Statistician, Cork University Hospital. Incidence of Tuberculosis Disease in Southern Ireland per 100,000 [95% CI] from 2003 to 2016)

The year-on-year incidence in the 20 to 35-year-old age range does not decrease as one would expect, but this is due to the small number of cases in region A and B. (Fig. 2).

### Limitations

This study has a number of limitations. The unvaccinated population in Region-C represents an urban area. While population density was accounted for, deprivation index was not. This needs to be considered as a potential confounding factor, as homelessness and lower socioeconomic classes are traditionally considered greater issues in urban areas.

Population rather than individual BCG vaccination coverage estimates were used. Uptake of vaccines in the different policy areas was not represented in the data. It was reported that several children where there was no BCG vaccination available (Region-C) received the BCG vaccine in other jurisdictions due to parental concern following local outbreaks.

The small number of cases, and sample size make it more difficult to detect any substantial decrease in the incidence rate over time, particularly when attempting to focus on a specific age group i.e. the 25–35-year olds.

Finally, the study period began after implementation of the differing vaccine policies and thus it is not possible to directly assess the effect of each policy on each region.

### Strengths

In this study we observe the impact of different BCG policies on a defined geographic region over time. Although this is widely studied across different resource settings and countries, to our knowledge this has not been studied on populations with such comparable demographics in a low TB disease incidence country.

## Discussion

Prevention and treatment of TB remains a significant challenge worldwide. In 2017, the WHO estimated that 23% of the world’s population have latent TB infection (LTBI) and that there had been 1.6 million TB disease related deaths [1]. In Ireland in 2017 the crude national incidence rate of TB disease was 6.7/100,000; however there is little information in relation to the prevalence of LTBI in Ireland [13].

National data from the HPSC shows that the incidence of TB disease in Southern Ireland during the study period was consistently above that of the general Irish population [11]. We postulate that this is as a result of the variation in regional BCG vaccine policy.

Presently, there are three health interventions available for TB prevention; screening and proactive treatment of LTBI, transmission prevention through infection control and targeted vaccination of at-risk groups with the BCG vaccine.

BCG vaccine is no longer recommended in the general population in low incidence countries including Ireland as its use is not supported by risk-benefit or cost effectiveness analysis [14, 15]. In low incidence countries prevention strategies focus on screening at-risk groups and chemoprophylaxis with BCG vaccine targeted to specific at-risk populations. BCG vaccination programmes continue in high incidence countries.

## Conclusion

Our study demonstrates significant regional variation in the incidence of TB in demographically similar populations based on BCG vaccination policy. This observation is particularly noteworthy in a country with low TB disease incidence such as Ireland. These findings strengthen existing data demonstrating efficacy of BCG vaccination for primary prevention of TB disease.

## Additional file

Additional file 1: Cluster of 138 cases of Tuberculosis Disease in unvaccinated population Numbers plotted: Relative risk compared to the area outside the cluster [95% CI]. 138 cases in a population of 46,000 were identified by spatial cluster analysis of all cases identified throughout the study period. Abbreviations: RR Relative Risk. Attribution: Dr. Darren Dahly, Principal Statistician, Cork University Hospital. (PNG 1120 kb)

## Abbreviations

BCG: Bacille Camille-Guerin
HPSC: Health Protection Surveillance Centre
HSE: Health Service Executive
LHO: Local Health Offices
LTBI: Latent TB infection
MTB: *Mycobacterium tuberculosis*
TB: Tuberculosis
WHO: World Health Organisation
