# Supplementary figures and images for: Impact of BCG vaccination on incidence of tuberculosis disease in southern Ireland
Source: BMC Infect Dis. 2019 May 9;19:397. doi: 10.1186/s12879-019-4026-z (PMC6506945; doi:10.1186/s12879-019-4026-z)

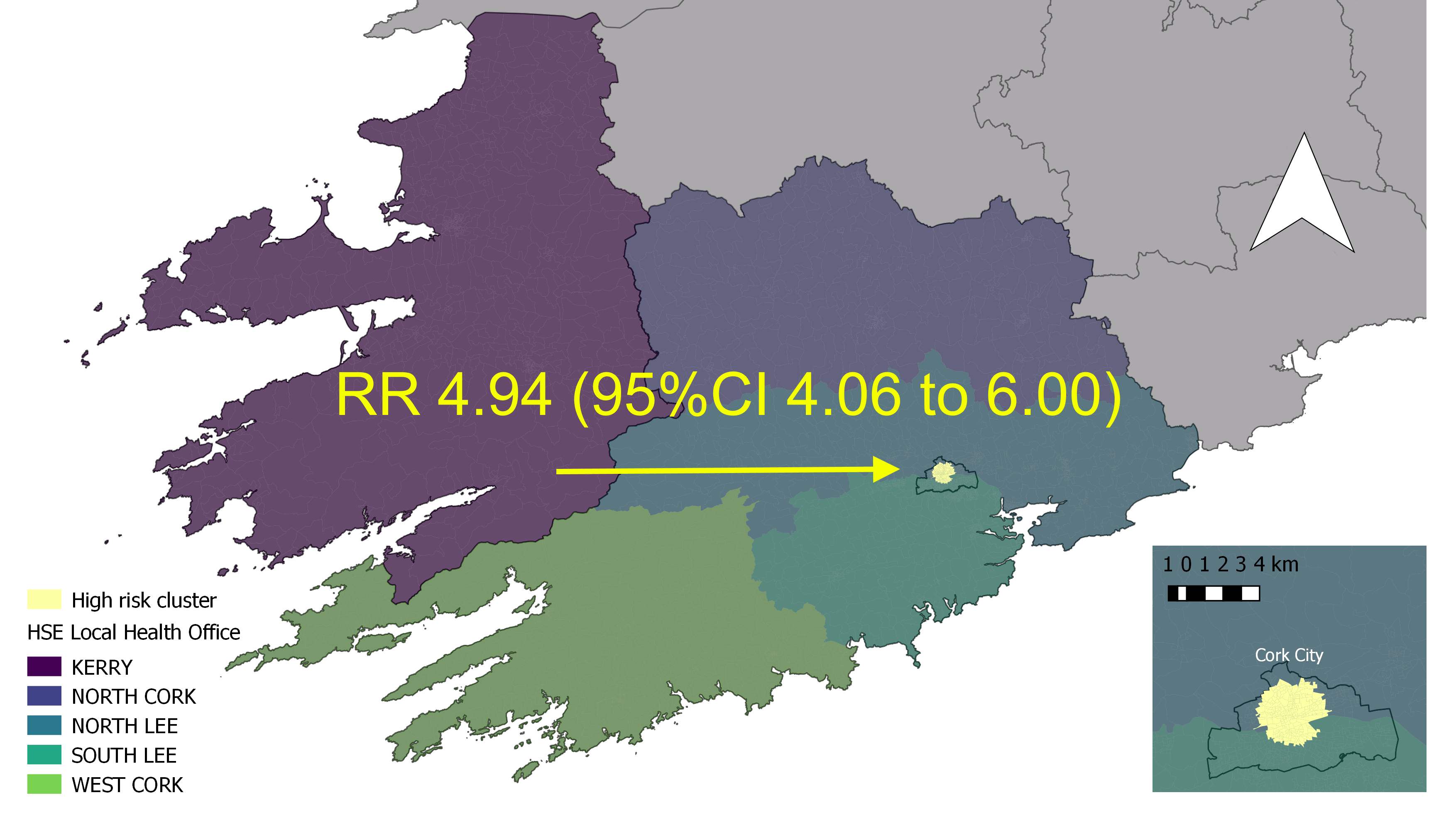

Supplement: Supplementary file 1 — Cluster of 138 cases of Tuberculosis Disease in unvaccinated population Numbers plotted: Relative risk compared to the area outside the cluster [95% CI]. 138 cases in a population of 46,000 were identified by spatial cluster analysis of all cases identified throughout the study period. Abbreviations: RR Relative Risk. Attribution: Dr. Darren Dahly, Principal Statistician, Cork University Hospital. (PNG 1120 kb) [file 12879_2019_4026_MOESM1_ESM.png]
